# Moderate or severe LUTS is associated with increased recurrence of non - muscle - invasive urothelial carcinoma of the bladder

**DOI:** 10.1590/S1677-5538.IBJU.2018.0068

**Published:** 2019-04-01

**Authors:** Austin Lunney, Allan Haynes, Pranav Sharma

**Affiliations:** 1 Department of Urology, Texas Tech University Health Sciences Center, Lubbock, TX, USA

**Keywords:** Urinary Bladder Neoplasms, Lower Urinary Tract Symptoms, Carcinoma, Transitional Cell

## Abstract

**Purpose:**

Non - muscle - invasive bladder cancer (NMIBC) can recur despite transurethral resection (TURBT) and adjuvant intravesical therapy. Tobacco products excreted in the urine are hypothesized to cause tumor - promoting effects on urothelial cells through direct contact. We determined if moderate or severe lower urinary tract symptoms (LUTS) (defined as International Prostate Symptom Score [IPSS] ≥ 8) was associated with increased tumor recurrence.

**Materials and Methods:**

We retrospectively identified 70 consecutive men initially diagnosed with NMIBC at our institution from 2010 – 2016. Means were compared with independent T - test and proportions with chi - square analysis. Multivariate logistic regression was performed to determine independent predictors of recurrence.

**Results:**

The majority of patients had Ta disease (58.6%) followed by T1 (28.6%) and Tis (12.9%). Forty - one (58.6%) patients had moderate or severe LUTS upon presentation within 30 days of initial TURBT with mean IPSS of 13.2 vs. 5.2 in the control group (p < 0.01). Biopsy - proven tumor recurrence occurred in 24 (34.3%) patients at mean follow-up of 31.7 months. Mean time to recurrence was 14.6 months. Moderate or severe LUTS was an independent predictor of tumor recurrence (odds ratio [OR]: 19.1, 95% confidence interval [CI]: 2.86 – 127; p = 0.002). Voiding or storage symptoms based on the IPSS did not independently correlate with tumor recurrence (p = 0.08 and p = 0.31, respectively) although total mean IPSS score did (OR: 1.26, 95% CI: 1.07 – 1.47, p = 0.005).

**Conclusions:**

The presence of moderate or severe LUTS may be an important prognostic factor in NMIBC. Patients with significant urinary symptoms could be monitored more aggressively due to higher recurrence risk.

## INTRODUCTION

Approximately 75% of bladder cancer patients present with non - muscle invasive disease classified as Ta (mucosa only), T1 (lamina propria invasion), or carcinoma in situ (CIS) (1, 2). Non - muscle invasive bladder cancer (NMIBC) has a high rate of recurrence and progression despite local treatment (3). Recurrence rates range from < 20% for low - grade, Ta lesions to 80% for high - grade, T1 tumors (4). Probabilities for recurrence are dependent on tumor size, grade, stage, multiplicity, and initial response to intravesical therapy (5).

Risk factors for urothelial carcinoma (UC) include tobacco abuse, which is estimated to account for 50% of tumors (6). It is hypothesized that tobacco products exert a tumor - promoting effect on the urothelial cells of the bladder mucosa through direct contact with the urine via mechanisms such as immunomodulation (7).

Benign prostatic hypertrophy (BPH) is common in men and can cause bladder outlet obstruction (BOO), which can result in lower urinary tract symptoms (LUTS) such as frequency, urgency, and hesitancy (8). Retained urine secondary to BPH - related LUTS can theoretically lead to increased exposure to urinary carcinogens and higher recurrence rates. In this study, we determined if moderate or severely symptomatic LUTS at the time of diagnosis was associated with greater tumor recurrence in male patients being treated for NMIBC.

## MATERIALS AND METHODS

### Data Collection

After institutional review board approval, we retrospectively identified 70 male patients with newly - diagnosed NMIBC at our institution between January 2010 and December 2016. Pathology was confirmed by central histopathological review. We excluded patients with non - pure urothelial carcinoma of the bladder, muscle - invasive or metastatic disease (confirmed through pathology or radiographic imaging), prior history of urothelial carcinoma of the urinary tract, or concurrent or prior tumors outside of the bladder (upper tracts, prostate, urethra). Patients re - staged to muscle - invasive disease after repeat transurethral resection (TURBT) were not included.

Socio - demographics and comorbidity indicators (Charlson Comorbidity Index [CCI]) were abstracted from the initial Urology clinic visit. International Prostate Symptom Score (IPSS), a standardized, validated screening tool to self - report bothersome urinary symptoms, was completed by all patients at the initial visit. IPSS consisted of seven questions regarding urinary bother, which were subcategorized into questions dealing with voiding symptoms (incomplete emptying, intermittency, weak stream, and straining to void) and those dealing with storage symptoms (frequency, urgency, and nocturia), as well as an overall quality of life (QoL) score. Moderate or severe LUTS was defined as an IPSS ≥ 8. American Society of Anesthesiologists (ASA) score was assessed at time of TURBT, and follow-up was defined from time of initial TURBT until date of last contact or death. Tumor size was estimated based on the area of resection from the operative note, and for multifocal tumors, the size of the largest lesion treated was used. Pathological stage and grade of the most aggressive tumor on initial or re - resection TURBT was assigned with CIS considered to be the most aggressive stage for NMIBC.

### Clinical management and follow-up

Patients were treated according to NCCN bladder cancer guidelines for NMIBC (9). Baseline upper tract imaging was performed on all patients with CT urogram, non - contrast CT scan, or renal ultrasound and bilateral retrograde pyelograms in the presence of renal dysfunction. Initial TURBT was completed within 60 days of presentation to the Urology clinic, and bladder tumors were completed resected on initial or repeat TURBT based on subjective intraoperative review. Utilization of post - resection mitomycin C was variable, but patients with incomplete initial TURBT, high - grade bladder tumors, or T1 tumors (with or without muscularis propria present in the initial specimen) underwent re - resection within 4 – 6 weeks for accurate staging. Ureteral washings, prostatic urethral biopsies, or random bladder biopsies were not routinely performed at the time of initial or repeat TURBT, and blue light cystoscopy was not available, so all procedures were done under white light.

Patients with low - grade, Ta bladder tumors were not routinely treated with adjuvant intravesical chemotherapy and were followed with observation. Patients with high - grade, T1, or CIS tumors, however, received at least one six - week induction cycle of adjuvant intravesical Bacillus Calmette - Guérin (BCG) 2 – 4 weeks after initial or repeat TURBT.

Follow-up after TURBT and adjuvant intravesical therapy (if indicated) consisted of clinic cystoscopy and urine cytology every 3 months for the first year, every 6 months for the second year, and annually thereafter indefinitely. Upper tract imaging was performed every 1 – 2 years. We did not routinely perform biopsies after adjuvant intravesical therapy to assess for tumor response unless a clinical suspicion for recurrence arose based on clinic cystoscopy, urine cytology, upper tract imaging, or new - onset gross hematuria. Recurrence was based on definitive diagnosis from histopathology from repeat TURBT or biopsy of the urinary tract during the surveillance period.

### Statistical Analysis

Sociodemographic, comorbidity, and other relevant clinical variables were compared between patients based on severity of LUTS and our primary endpoint, which was the development of a pathology - proven recurrence in the urinary tract during follow-up. Continuous variables were reported as means and standard deviations (SD) and categorical variables as frequency counts and percentages. We used the independent T - test to determine any differences in continuous variables and the chi - square test for categorical variables. Multivariate logistic regression analysis was performed to evaluate the association of recorded variables with our primary endpoint, and odds ratios (OR) were reported. Factors analyzed included any variable with a statistically significant association with our primary endpoint on bivariate analysis. Statistical analysis were performed with the Statistical Package for the Social Sciences (SPSS) software package (IBM Corporation, Armonk, NY). All tests were 2 - sided, with p < 0.05 considered to be statistically significant.

## RESULTS

The majority of patients had small (< 2.0 cm) (54.3%), solitary (61.4%) tumors and did not receive post - resection mitomycin C (75.7%). Ta disease was the most predominant (58.6%) followed by T1 (28.6%) and Tis (12.9%), and the majority of tumors were high - grade (55.7%).

Moderate or severe LUTS was seen in 41 (58.6%) men upon presentation to the Urology clinic within 30 days of initial TURBT. These patients had a mean IPSS of 13.2 compared to 5.2 in those with mild LUTS (p < 0.01). Patients with moderate or severe LUTS were more likely to be older (mean age: 69.2 vs. 59.5 years, p < 0.01), on BPH medications (78% vs. 17.2%, p < 0.01), and have high - grade tumors (70.7% vs. 34.5%, p = 0.003) (Table-1). The most common class of BPH medications taken by our study population were alpha - blockers (n = 24; 64.9%) followed by 5 - alpha - reductase inhibitors (n = 11; 29.7%) and phosphodiesterse – 5 inhibitors (n = 2; 5.4%).

**Table 1:** Patient characteristics and outcomes based on the degree of LUTS.

		Mild LUTS [IPSS < 8] (n = 29)	Moderate or severe LUTS [IPSS > 8] (n = 41)	Total (n = 70)	p - value
**Sociodemographic and clinical characteristics**					
	Mean age, years (SD)	59.5 (10.9)	69.2 (10.5)	65.2 (11.6)	< 0.01
	Mean BMI, kg / m2 (SD)	30.7 (7.4)	29.1 (6.3)	29.8 (6.8)	0.35
**Race, no. (%)**					**0.34**
	White	23 (79.3)	36 (87.8)	59 (84.3)	
	Non - white	6 (20.7)	5 (12.2)	11 (15.7)	
**Tobacco abuse, no. (%)**					**0.51**
	None	15 (51.7)	16 (39.0)	31 (44.3)	
	Former	5 (17.2)	11 (26.8)	16 (22.9)	
	Current	9 (31.0)	14 (34.1)	23 (32.9)	
**Family History of UC, no. (%)**					**0.72**
	No	27 (93.1)	39 (95.1)	66 (94.3)	
	Yes	2 (6.9)	2 (4.9)	4 (5.7)	
**Charlson Comorbidity Index, no. (%)**					**0.49**
	≤ 4	19 (65.5)	21 (51.2)	40 (57.1)	
	5 – 7	9 (31.0)	19 (43.9)	27 (38.6)	
	≥ 8	1 (3.4)	2 (4.9)	3 (4.3)	
**ASA Score, no. (%)**					**0.26**
	≤ 2	13 (44.8)	13 (31.7)	26 (37.1)	
	≥ 3	16 (55.2)	28 (68.3)	44 (62.9)	
**BPH medications, no. (%)**					**<0.01**
	No	24 (82.8)	9 (22.0)	33 (47.1)	
	Yes	5 (17.2)	32 (78.0)	37 (52.9)	
**Disease - specific characteristics**					
**Tumor size, no. (%)**					**0.39**
	Small (0.5 – 2.0 cm)	19 (65.5)	19 (46.3)	38 (54.3)	
	Medium (2.0 – 5.0 cm)	8 (27.6)	16 (39.0)	26 (37.1)	
	Large (> 5.0 cm)	2 (6.9)	4 (9.8)	6 (8.6)	
**Tumor focality, no. (%)**					**0.93**
	Solitary	18 (62.1)	25 (61.0)	43 (61.4)	
	Multiple	11 (37.9)	16 (39.0)	27 (38.6)	
**Pathological Tumor Grade, no. (%)**					**0.003**
	Low - grade	19 (65.5)	12 (29.3)	31 (44.3)	
	High - grade	10 (34.5)	29 (70.7)	39 (55.7)	
**Pathological Tumor Stage, no. (%)**					**0.41**
	Ta	19 (65.5)	22 (53.7)	41 (58.6)	
	T1	8 (27.6)	12 (29.3)	20 (28.6)	
	Tis	2 (6.9)	7 (17.1)	9 (12.9)	
**Post - resection Mitomycin C, no. (%)**					**0.98**
	No	22 (75.9)	31 (75.6)	53 (75.7)	
	Yes	7 (24.1)	10 (24.4)	17 (24.3)	
**Pathology - proven recurrence, no. (%)**					**< 0.01**
	No	27 (93.1)	19 (46.3)	46 (65.7)	
	Yes	2 (6.9)	22 (53.7)	24 (34.3)	

Twenty - four patients (34.3%) experienced a pathology - proven recurrence in the urinary tract. Mean follow-up was 31.7 months, and mean time to first recurrence was 14.6 months. Three patients (4.3%) had a recurrence on their first surveillance cystoscopy at 3 months. The majority of recurrences were within the urinary bladder (n = 23) with one occurring superficially in the prostatic urethra. Twenty - two of 41 (53.7%) patients with moderate or severe LUTS developed a tumor recurrence during follow - up versus 2 of 29 (6.9%) with mild LUTS (Table-1). Tumor progression occurred in four patients with three having pathological tumor upstaging and one developing muscle - invasive disease.

Patients who experienced a recurrence had higher overall urinary bother (mean IPSS: 13.3 vs. 8.2, p < 0.01) including more voiding symptoms (mean IPSS voiding: 7.9 vs. 5.0, p < 0.01) and storage symptoms (mean IPSS storage: 5.4 vs. 3.2, p < 0.01), and worse urinary QoL (mean IPSS QoL: 3.8 vs. 2.5, p < 0.01) (Table-2). These patients also had more high - grade tumors (79.2% vs. 43.5%, p = 0.004) and advanced tumor stage (p = 0.001) (Table-2).

**Table 2:** Patient characteristics and outcomes stratified by the presence of a recurrence.

		No recurrence (n = 46)	Recurrence (n = 24)	Total (n = 70)	p - value
**Sociodemographic and clinical characteristics**					
	Mean age, years (SD)	63.8 (11.4)	67.8 (11.8)	65.2 (11.6)	0.18
	Mean BMI, kg / m2 (SD)	29.8 (7.1)	29.9 (6.2)	29.8 (6.8)	0.95
**Race, no. (%)**					**0.22**
	White	37 (80.4)	22 (91.7)	59 (84.3)	
	Non - white	9 (19.6)	2 (8.3)	11 (15.7)	
**Tobacco abuse, no. (%)**					**0.29**
	None	18 (39.1)	13 (54.2)	31 (44.3)	
	Former	10 (21.7)	6 (25.0)	16 (22.8)	
	Current	18 (39.1)	5 (20.8)	23 (32.9)	
**Family History of UC, no. (%)**					**0.69**
	No	43 (93.5)	23 (95.8)	66 (94.3)	
	Yes	3 (6.5)	1 (4.2)	4 (5.7)	
**Charlson Comorbidity Index, no. (%)**					**0.93**
	≤ 4	27 (58.7)	13 (54.2)	40 (57.1)	
	5 – 7	17 (37.0)	10 (41.7)	27 (38.6)	
	≥ 8	2 (4.3)	1 (4.2)	3 (4.3)	
**ASA Score, no. (%)**					**0.96**
	≤ 2	17 (37.0)	9 (37.5)	26 (37.1)	
	≥ 3	29 (63.0)	15 (62.5)	44 (62.9)	
**BPH medications, no. (%)**					**0.09**
	No	25 (54.3)	8 (33.3)	33 (47.1)	
	Yes	21 (45.7)	16 (66.7)	37 (52.9)	
Mean IPSS (SD)		8.2 (4.6)	13.3 (4.9)	9.9 (5.3)	< 0.01
Mean IPSS QoL (SD)		2.5 (1.4)	3.8 (1.0)	3.0 (1.4)	< 0.01
Mean IPSS Voiding (SD)		5.0 (2.8)	7.9 (2.6)	6.0 (3.1)	< 0.01
Mean IPSS Storage (SD)		3.2 (2.2)	5.4 (2.8)	3.9 (2.6)	< 0.01
**Disease - specific characteristics**					
**Tumor size, no. (%)**					**0.51**
	Small (0.5 – 2.0 cm)	27 (58.7)	11 (45.8)	38 (54.3)	
	Medium (2.0 – 5.0 cm)	16 (34.8)	10 (41.7)	26 (37.1)	
	Large (> 5.0 cm)	3 (6.5)	3 (12.5)	6 (8.6)	
**Tumor focality, no. (%)**					**0.89**
	Solitary	28 (60.9)	15 (62.5)	43 (61.4)	
	Multiple	18 (39.1)	9 (37.5)	27 (38.6)	
**Pathological Tumor Grade, no. (%)**					**0.004**
	Low - grade	26 (56.5)	5 (20.8)	31 (44.3)	
	High - grade	20 (43.5)	19 (79.2)	39 (55.7)	
**Pathological Tumor Stage, no. (%)**					**0.001**
	Ta	34 (73.9)	7 (29.2)	41 (58.6)	
	T1	9 (19.6)	11 (45.8)	20 (28.6)	
	Tis	3 (6.5)	6 (25.0)	9 (12.9)	
**Post - resection Mitomycin C, no. (%)**					**0.28**
	No	33 (71.7)	20 (83.3)	53 (75.7)	
	Yes	13 (28.3)	4 (16.7)	17 (24.3)	

On multivariate analysis, the presence of moderate or severe LUTS was an independent predictor of tumor recurrence (OR: 19.1, 95% confidence interval [CI]: 2.86 – 127; p = 0.002) in addition to pathological tumor stage (p < 0.05) (Table-3). Voiding or storage symptoms based on the IPSS did not independently correlate with tumor recurrence in our multivariate model (p = 0.08 and p = 0.31, respectively) (Table-4) although total mean IPSS score did (OR: 1.26, 95% CI: 1.07 – 1.47, p = 0.005) (Table-5).

**Table 3:** Predictors of pathology - proven tumor recurrence in the urinary tract based on the degree of LUTS.

		Multivariate			
		OR	95% CI		p - value
			Lower	Upper	
Mean age, years		1.01	0.95	1.08	0.75
Moderate or severe LUTS [IPSS ≥ 8] (reference: mild LUTS [IPSS < 8])		19.1	2.86	127	0.002
High - grade tumor (reference: low - grade)		1.06	0.22	5.02	0.95
**Pathological Tumor Stage**					
	pT1 tumor (reference: pTa)	9.91	1.74	56.4	0.01
	pTis tumor (reference: pTa)	10.4	1.32	82.3	0.026

**Table 4:** Predictors of pathology - proven tumor recurrence in the urinary tract based on IPSS subcategories.

		Multivariate			
		OR	95% CI		p - value
			Lower	Upper	
Mean age, years		1.01	0.95	1.08	0.75
Mean IPSS Voiding		1.30	0.97	1.74	0.08
Mean IPSS Storage		1.20	0.84	1.71	0.31
High - grade tumor (reference: low - grade)		1.36	0.31	6.11	0.69
**Pathological Tumor Stage**					
	pT1 tumor (reference: pTa)	7.35	1.41	38.4	0.018
	pTis tumor (reference: pTa)	10.6	1.47	76.9	0.019

**Table 5:** Predictors of pathology - proven tumor recurrence in the urinary tract based on overall IPSS.

		Multivariate			
		OR	95% CI		p value
			Lower	Upper	
Mean age, years		1.01	0.95	1.08	0.75
Mean IPSS		1.26	1.07	1.47	0.005
High - grade tumor (reference: low - grade)		1.39	0.31	6.14	0.67
**Pathological Tumor Stage**					
	pT1 tumor (reference: pTa)	7.57	1.48	38.8	0.015
	pTis tumor (reference: pTa)	10.4	1.44	75.1	0.020

## DISCUSSION

In this retrospective, cohort study of male patients with NMIBC, there was an association between the presence of moderate to severe LUTS on presentation (defined as an initial IPSS ≥ 8) with the risk of initial tumor recurrence on follow-up. Higher mean IPSS also correlated with an increased incidence of recurrence but subcategorization based on voiding versus storage symptoms did not show that one set was more strongly related to recurrence risk.

We hypothesis this association may be related to increased urinary contact time of the bladder mucosa in patients with voiding dysfunction secondary to incomplete bladder emptying. The urogenous contact hypothesis was first introduced in the mid – 1970s and claims there is a connection between urinary contact time and the development of urothelial carcinoma of the bladder (10). In a study that looked at the association between fluid intake and the risk of bladder cancer in men, investigators analyzed 47.909 men and the amount of fluid they consumed (11). The authors determined that a higher fluid intake was associated with a reduced risk of bladder cancer. The authors hypothesized that this inverse relationship between fluid intake and bladder cancer was secondary to reduced urinary contact time with the bladder and less exposure to potentially harmful carcinogens. Silverman et al. additionally looked at the association between nighttime voiding and bladder cancer (10). This study similarly found an inverse relationship between nocturia and bladder cancer, stating that men and women who voided at least twice per night experienced a significant reduction in risk of malignancy. The authors hypothesized that those who void less at night have increased urinary contact time and thus a higher risk of bladder cancer.

In the typical age group that men develop bladder cancer (60 – 80 years), BPH can cause significant LUTS, such as urinary frequency, urgency, and hesitancy because of obstruction of the bladder. This can cause elevated post void residual (PVR) urine from incomplete bladder emptying, which may increase urinary contact time and exposure to carcinogens in the urine. Zhou et al. examined the risk of bladder cancer in relation to the severity of LUTS among 30.183 men from 1996 until 2010 using data from the Health Professionals Follow-up Study (12). Men with severe LUTS had a 64% higher risk of bladder cancer (relative risk [RR]: 1.64; 95% CI: 0.87 – 3.08) compared to men with no reported LUTS. Patients with both voiding and storage dysfunction had a significantly higher risk of bladder cancer (RR: 1.60; 95% CI: 1.00 – 2.56), and urinary hesitancy was the strongest individual urinary symptom associated with bladder cancer (RR: 2.21; 95% CI: 1.29 – 3.78).

Matsumoto et al. showed in an animal model that when rats have a surgically induced partial bladder outlet obstruction and a carcinogen is introduced into the water, there is a greater incidence of bladder cancer (13). No bladder cancer was found in the rats exposed to the carcinogen alone, but six out of the ten rats with partial bladder outlet obstruction and exposure to the carcinogen developed bladder cancer after eight weeks. Tseng et al. also reported that BPH was a significant independent risk factor for bladder cancer in men with type 2 diabetes in Taiwan using national reimbursement data from 1996 to 2009 (8). The incidences of bladder cancer were 258.77 and 69.34 per 100.000 person - years for patients with and without BPH, respectively.

Early resolution of BOO and lower PVR volume may decrease the recurrence rate of bladder tumors secondary to lower urinary contact time. Ham et al. observed that tumor recurrence was significantly lower, and time to recurrence was longer after simultaneous TURBT and transurethral resection of the prostate (TURP) in men with concurrent urothelial carcinoma of the bladder and BOO secondary to BPH causing LUTS (14). The 60 - month recurrence - free probability was 52% compared to 43% (p < 0.01) in patients who had TURBT alone without any resolution of their BPH / BOO. There was no difference in progression rates between the two groups. A similar study from Europe corroborated this finding, showing a reduced 5 - year recurrence rate (56% vs. 80%, p < 0.01) in men diagnosed with Tis, Ta, or T1 urothelial carcinoma of the bladder and BPH / BOO who underwent TURBT and TURP in the same setting versus TURBT alone (15). Early resolution of bladder outlet obstruction not only decreased tumor recurrence rate, but also had a positive effect on patient quality of life. Two meta - analysis have additionally reinforced suggestions that simultaneous TURBT and TURP may be beneficial in reducing tumor recurrence in patients with both superficial urothelial carcinoma of the bladder and BOO secondary to BPH. Luo et al. evaluated pooled data from 483 patients in six eligible clinical trials and found that the recurrence rate in the simultaneous resection group was statistically significantly lower than in the control group (TURBT alone) (OR = 0.67; 95% CI = 0.52 to 0.88, p = 0.003) (16). Li et al. also reported on pooled data from eight studies, including seven non - randomized concurrent controlled trials and one randomized controlled trial, involving a total of 1.372 patients (17). Meta - analyses showed that in the TURBT + TURP group, overall recurrence rates were lower (OR = 0.76; 95% CI = 0.60 – 0.96; p = 0.02), and this difference was statistically significant. The postoperative recurrence rate in the prostatic fossa / bladder neck (OR = 0.96; 95% CI = 0.64 – 1.45; p = 0.86) and bladder tumor progression rates (OR = 0.96; 95% CI = 0.49 – 1.87; p = 0.91) were similar between the TURBT + TURP and TURBT groups, and the difference was not significant. Both sets of authors, therefore, concluded that for patients with NMIBC and BPH / BOO, simultaneous resection may reduce the recurrence rate. Combined TURBT and TURP may be a reasonable option in NMIBC patients who present with significant urinary symptoms.

Our study does have several limitations. It is underpowered with only 70 male patients with NMIBC in our study population with limited mean follow-up of only 31.7 months. We were, however, very selective in our inclusion criteria to minimize confounding, excluding patients with histological variants, prior history of urothelial carcinoma, and non - bladder primary tumors.

Additionally, we did not have PVR volumes available via bladder scan or post - void catheterization for our study population. We were, therefore, unable to objectively measure the degree of incomplete bladder emptying across our study population for male patients with varying levels of LUTS, and we could not correlate these volumes with the recurrence rate of urothelial carcinoma. Only subjective measures of voiding dysfunction based on patient self - reported IPSS were abstracted.

Finally, given the study's retrospective design, there is the possibility of bias via inverse causality. In other words, men who present with NMIBC and bothersome urinary symptoms had a higher grade and stage of tumor that would be more prone to recurrence due to worse underlying disease rather than ongoing urinary symptoms causing the recurrence.

## CONCLUSIONS

In this study, we reported that the presence of moderate or severely symptomatic LUTS in men at the time of diagnosis is associated with increased recurrence rates of NMIBC based on surveillance cystoscopy, imaging, and histological confirmation. Further evaluation of this hypothesis with objective measurements of voiding dysfunction such as PVR volumes should be considered. Additional prospective studies with larger sample sizes and longer term follow-up are necessary to corroborate these findings.

## ABBREVIATIONS

ASA: = American Society of Anesthesiologists
BCG: = Bacillus Calmette - Guérin
BOO: = bladder outlet obstruction
BPH: = benign prostatic hyperplasia
CCI: = Charlson Comorbidity Index
CI: = confidence interval
CIS: = carcinoma in situ
IPSS: = International Prostate Symptom Score
LUTS: = lower urinary tract symptoms
NMIBC: = non - muscle invasive bladder cancer
OR: = odds ratio
PVR: = post void residual
QoL: = quality of life
RR: = relative risk
SPSS: = Statistical Package for the Social Sciences
TURBT: = transurethral resection of bladder tumor
TURP: = transurethral resection of the prostate
UC: = urothelial carcinoma
